# Genome-Wide Association Study Reveals Candidate Genes for Control of Plant Height, Branch Initiation Height and Branch Number in Rapeseed (*Brassica napus* L.)

**DOI:** 10.3389/fpls.2017.01246

**Published:** 2017-07-18

**Authors:** Ming Zheng, Cheng Peng, Hongfang Liu, Min Tang, Hongli Yang, Xiaokang Li, Jinglin Liu, Xingchao Sun, Xinfa Wang, Junfeng Xu, Wei Hua, Hanzhong Wang

**Affiliations:** ^1^Key Laboratory of Biology and Genetic Improvement of Oil Crops, Ministry of Agriculture, Oil Crops Research Institute of the Chinese Academy of Agricultural Sciences Wuhan, China; ^2^State Key Laboratory Breeding Base for Zhejiang Sustainable Pest and Disease Control, Zhejiang Academy of Agricultural Sciences Hangzhou, China

**Keywords:** *Brassica napus*, plant height, branch initiation height, branch number, GWAS, QTLs

## Abstract

Plant architecture is crucial for rapeseed yield and is determined by plant height (PH), branch initiation height (BIH), branch number (BN) and leaf and inflorescence morphology. In this study, we measured three major factors (PH, BIH, and BN) in a panel of 333 rapeseed accessions across 4 years. A genome-wide association study (GWAS) was performed via Q + K model and the panel was genotyped using the 60 k *Brassica* Infinium SNP array. We identified seven loci for PH, four for BIH, and five for BN. Subsequently, by determining linkage disequilibrium (LD) decay associated with 38 significant SNPs, we gained 31, 15, and 17 candidate genes for these traits, respectively. We also showed that PH is significantly correlated with BIH, while no other correlation was revealed. Notably, a GA signaling gene (*BnRGA*) and a flowering gene (*BnFT*) located on chromosome A02 were identified as the most likely candidate genes associated with PH regulation. Furthermore, a meristem initiation gene (*BnLOF2*) and a NAC domain transcriptional factor (*BnCUC3*) that may be associated with BN were identified on the chromosome A07. This study reveals novel insight into the genetic control of plant architecture and may facilitate marker-based breeding for rapeseed.

## Introduction

Rapeseed (*Brassica napus* L., AACC, 2n = 38) is a globally significant oilseed crop used in the production of vegetable oil and oil meals, and ~95% of its total cultivation is in Asia, Europe, and North America. According to a new report from the United States Department of Agriculture (USDA), rapeseed production in 2015/16 is estimated at 66.9 million tons, down 4.7% from the previous year. Harvested area is estimated at 32.9 million hectares, down 5.5% from the previous year (http://www.pecad.fas.usda.gov/). To meet growing demands for both edible products and biofuel, it is critical to increase rapeseed productivity through efficient breeding.

Rapeseed productivity is determined by three components: number of siliques per plant (NSP), number of seeds per silique (NSS), and seed weight per plant (Clarke and Simpson, 1978). Simultaneously, rapeseed plant architecture is a crucial determinant of its yield and is itself determined by plant height (PH) and branch number, as well as inflorescence morphology, which indirectly influences its yield by affecting NSP (Qiu et al., 2006; Chen et al., 2014). Plant height was first selected to improve crop yields: taller plants were more easily affected by lodging and therefore showed decreased yield, while dwarf plants showed resistance to lodging and their yield could be further increased by the use of nitrogen fertilizers. In the late 1960s and early 1970s, the dwarf gene was rapidly adopted in wheat and rice to cultivate new varieties, which greatly increased their yield and precipitated the “Green Revolution” (Peng et al., 1999; Evenson and Gollin, 2003). In addition, most domesticated crops also contain the plant domestication gene *TB1* (Doebley et al., 1997; Takeda et al., 2003; Aguilar-Martinez et al., 2007; Lewis et al., 2008). Recently, ideal plant architecture (IPA) has been proposed as a plant type with increased yield potential (Jiao et al., 2010; Miura et al., 2010).

In Arabidopsis and rice, the mechanisms regulating PH are well-known, and many phytohormones, including Gibberellins (GAs), Brassinosteroids (BRs), and Strigolactones (SLs) participate in this process. Genes related to phytohormone biosynthesis and signal transduction contain mutations that affect internode elongation, which in turn regulates PH (Ikeda et al., 2001; Ishikawa et al., 2005; Sun et al., 2010; Clouse, 2011; Jiang et al., 2013; Zhou et al., 2013). During the floral transition, axillary meristems are transformed into branch meristems to form branches (Teo et al., 2014). Phytohormone, including Auxin, Cytokinins (CKs), and SLs biosynthesis, transport, and signaling genes also play important roles in branch formation (Ferguson and Beveridge, 2009; Janssen et al., 2014), as do floral meristem identity genes (i.e., MYB family RAX proteins, ALOG family proteins, other meristem identity genes, flowering time genes, etc.; Liljegren et al., 1999; Hiraoka et al., 2013; Liu et al., 2013). In Arabidopsis, most integrated regulatory networks controlling the formation of branching are regulated by the gene *SHOOT MERISTEMLESS* (*STM*) (Long et al., 1996; Lenhard et al., 2002). Despite significant advances in other plants, our understanding of the molecular mechanisms of plant architecture regulation in rapeseed remains limited.

Rapeseed (*B. napus*) and the model plant Arabidopsis are members of the *Brassicaceae*. Unlike *Arabidopsis thaliana, B. napus* is polyphyletic, and is related to *B. rapa* (AA) and *B. oleracea* (CC) (Allender and King, 2010). So far, genomes have been sequenced for *B. rapa* (AA), *B. oleracea* (CC), and *B. napus* (AACC) (Wang et al., 2011; Chalhoub et al., 2014; Liu et al., 2014). *B. napus* is a young allopolyploid and most orthologous genes are duplicated compared to the respective progenitor genomes (Chalhoub et al., 2014). Consequently, obtaining a detailed characterization of each copy is difficult.

Genetic mapping studies have employed bi-parental mapping populations to identify many quantitative trait locus (QTLs) for yield traits in rapeseed. However, most of these QTLs are localized to a large interval (10–20 cM) on chromosomes (Butruille et al., 1999; Quijada et al., 2006; Udall et al., 2006; Chen et al., 2007; Mei et al., 2009; Shi et al., 2009). The genome-wide association study (GWAS) has become a powerful tool to identify multiple related candidate genes regulating important traits in crops (Huang et al., 2010; Brachi et al., 2011; Li H. et al., 2013). This method is performed by scanning a genome using abundant single-nucleotide polymorphisms (SNPs) representing broad genetic variability. Presently, the GWAS in rapeseed has been used to identify loci and candidate genes related to oil content, plant architecture, flowering time, and other yield traits (Schiessl et al., 2015; Li et al., 2016; Liu et al., 2016; Lu et al., 2016; Sun et al., 2016a,b; Sun F. M. et al., 2016; Wang et al., 2016; Xu et al., 2016).

In this study, we report a GWAS for plant architecture traits (PH, BIH, and BN) in rapeseed using the 60 K *Brassica* Infinium SNP array on a panel with 333 accessions that cover a broad range of genetic diversity. We further identify several possible candidate genes for the three traits underlying these QTLs by LD decays harboring significant SNPs. Although some candidate QTLs are consistent with those found in previous studies, we have also identified novel QTLs for these traits. Moreover, the SNPs and candidate genes in our study may facilitate marker-based breeding to improve plant architecture in order to increase rapeseed yield.

## Materials and methods

### Plant materials and trait measurement

The association population used in this study consisted of 333 diverse rapeseed accessions (20 are winter type, 308 are semi-winter type and 5 are unknown) collected as part of a recently published study (Sun F. M. et al., 2016). The rapeseed accessions were grown in two replicates over the course of 4 years (2012–2015) in Wuhan, China (E 114.32°, N 30.52°). Individuals from germplasm populations were genotyped using the 60 K *Brassica* Infinium SNP array. Accessions were grown in plots of 2 rows, with 10–15 plants in each row. Between eight and ten plants were selected from the center of each row to measure the three traits at maturity: PH, BIH, and BN.

The length of the plant from the base of the stem to the tip of the main inflorescence was noted as PH. The length from the base of the stem to the first primary branch base was recorded as BIH. We also measured the number of primary branches arising from the main shoot (BN). Correlation analyses of PH, BIH, and BN for the association panel were performed in R3.3.0 (Ihaka and Gentleman, 1996).

### *In silico* mapping and linkage disequilibrium (LD) analysis

The probe sequences of 52,157 SNPs were used to perform a BLASTN search through the *B. napus* genome database (Chalhoub et al., 2014). Only top blast-hits with an *e*-value threshold of *e*^−15^ were used. Some SNPs were excluded due to a match in BLAST with multiple loci with identical scores. SNPs that showed a minor allele frequency (MAF) of <5% or that showed a maximal missing frequency of >2% were excluded (Brachi et al., 2011), remaining 32,297 SNPs identified, covering all 19 chromosomes of the *B. napus* genome and providing approximately one SNP per 25 kb. An estimate of the linkage disequilibrium (LD) was calculated using squared allele frequency correlations (*r*^2^) between all pairs of SNPs using TASSEL 5.2.28 (Bradbury et al., 2007).

### Genome-wide association study

All the SNPs were used to calculate the principal component analysis (PCA) matrix using the GCTA tool, and a subset of 3,571 SNPs (MAF ≥ 0.2) evenly distributed across the whole genome (every 150 k) were selected to perform population structure (Q) and relative kinship analysis (K). STRUCTURE v2.3.4 was used to calculate a Q matrix, the putative number of genetic groups (*k*-value) setting from 1 to 10 with five independent runs (Pritchard et al., 2000). The length of the burn-in period and the number of Markov Chain Monte Carlo (MCMC) replications after burn-in were set to 50,000 and 100,000 iterations, respectively. The relative kinship K matrix was performed by the SPAGeDi software package (Hardy and Vekemans, 2002; Yang et al., 2011). The Δk method was used to determine the most likely number of groups or subpopulations, as described by Evanno et al. (2005).

Association analysis was performed using TASSEL 5.2.28 (Bradbury et al., 2007), using a mixed linear model (MLM) to calculate the association in all analyses, incorporating Q matrix/PCA and kinship data (K) (Zhao et al., 2011). The MLM was applied using default settings (P3D for variance component analysis and compression set to optimum level). For MLM (Q + K), the significance threshold for significantly associated markers was set to *p* ≤ 4.06 × 10^−4^ [−log_10_ (*p*-value) = 3.39].

### Candidate gene mining

To identify candidate genes, local LD decay was calculated using TASSEL5.2.28 to capture flanking regions of up to 450 kb on either side of significant SNPs, with a cut-off value of *r*^2^ = 0.2. Gene sequences that correspond to putative orthologs in *A. thaliana* were based on GO annotations (http://www.arabidopsis.org/index.jsp). The genes with GO terms for auxin, GA, IAA, SL, CK, and flowering time were highlighted, and the closest one of these genes was considered as the most likely candidate.

## Results

### Phenotypic variation among accessions for PH, BIH, and BN

**Figure 1:**
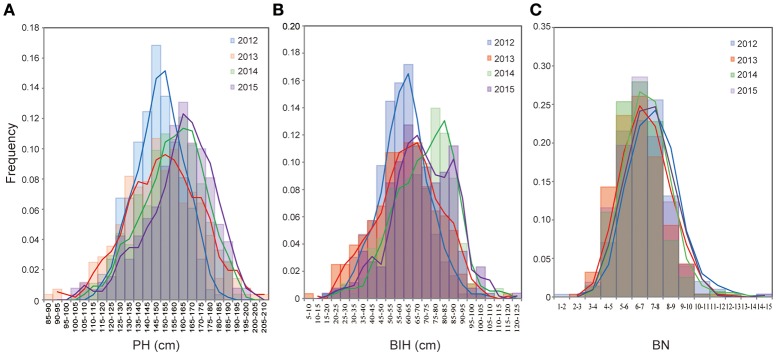
Frenquency distribution of PH **(A)**, BIH **(B)**, and BN **(C)** in the association panel of 333 accessions in 4 years (2012-2015). PH, plant height; BIH, branch initation height; BN, branch number.

Extensive phenotypic variations for PH, BIH, and BN were observed in the 333 accessions that were grown over the course of 4 years (2012–2015). PH varied from 86.2 to 206.0 cm, with 1.6 to 2.4-fold variations across the 4 years (Figure 1A, Table 1). BIH varied from 6.9 to 122.0 cm, with 5.9 to 17.3-fold variations across the 4 years (Figure 1B, Table 1). BN varied from 1.7 to 15.0, with 3.5 to 8.6-fold variations across the 4 years (Figure 1C, Table 1).

**Table 1:** Phenotypic variation of PH, BIH and BN in the association panel.

**Trait**	**Min**	**Max**	**Mean ± *SD***	**CV (%)**
PH-2012	113.6	182.2	147.4 ± 13.3	9.0
PH-2013	86.2	206.0	149.8 ± 20.7	13.8
PH-2014	106.3	192.4	154.9 ± 17.3	11.2
PH-2015	101.7	198.7	158.8 ± 18.3	11.5
BIH-2012	15.4	100.7	58.1 ± 13.5	23.2
BIH-2013	6.9	119.0	60.6 ± 18.0	29.6
BIH-2014	19.4	115.0	69.6 ± 16.3	23.5
BIH-2015	18.8	122.0	68.6 ± 17.2	25.0
BN-2012	1.7	14.3	6.9 ± 1.6	22.9
BN-2013	2.8	11.6	6.5 ± 1.5	22.7
BN-2014	3.7	12.9	6.6 ± 1.4	20.7
BN-2015	3.8	15.0	6.7 ± 1.5	21.9

*PH, plant height; BIH, branch initiation height; BN, branch number*.

In addition, we assessed these three traits for any significant correlation. PH and BIH were significantly correlated across the 4 years with a Pearson's correlation coefficient of 0.3 to 0.8 (Table S1). This suggests that these two traits may show genetic linkage or that some genes have pleiotropic roles in controlling these phenotypes. However, no other significant correlations were found in our accession panel, between the PH and BN, or between BN and BIH (Table S1).

### SNP quality control, performance, and *in silico* mapping

SNP genotyping was performed using the *Brassica* 60 K SNP array. We blasted the SNP probe sequences to the *B. napus* genome database (http://www.Genoscope.cns.fr/brassicanapus/) and a total of 34,292 SNP markers (65.7%) were selected to genotype the panel of 333 accessions (Table 2). The C04 linkage group had the most SNPs (3,182), with a marker density of one per 20.89 kb, whereas the C05 linkage group had the least SNPs (1,085), with a marker density of one per 39.8 kb (Table 2). Altogether, the overall mapping results demonstrate the high quality of the genotyping in this study.

**Table 2:** Number of SNPs and linkage disequilibrium (LD) decay on the 19 chromosomes of *B. napus*.

**Chromosome**	**Length (kb)**	**Number of SNP**	**SNP density (kb/SNP)**	**LD decay (kb)**
chrA01	23,267.86	1,676	13.88	130
chrA02	24,793.74	1,404	17.66	120
chrA03	29,767.49	2,444	12.18	110
chrA04	19,151.66	1,528	12.53	100
chrA05	23,067.60	1,658	13.91	150
chrA06	24,396.39	1,679	14.53	100
chrA07	24,006.52	1,855	12.94	100
chrA08	18,961.94	1,280	14.81	1,625
chrA09	33,865.34	1,676	20.21	420
chrA10	17,398.23	1,414	12.30	120
Subgenome A	238,676.76	16,614	14.37	150
chrC01	38,829.32	2,483	15.64	720
chrC02	46,221.80	2,169	21.31	1300
chrC03	60,573.39	2,900	20.89	425
chrC04	48,930.24	3,182	15.38	550
chrC05	43,185.23	1,085	39.80	240
chrC06	37,225.95	1,445	25.76	580
chrC07	44,770.48	1,715	26.11	1710
chrC08	38,477.09	1,596	24.11	640
chrC09	48,508.22	1,103	43.98	825
Subgenome C	406,721.72	17,678	23.01	700
A + C	645,398.48	34,292	18.82	450

### Linkage disequilibrium

**Figure 2:**
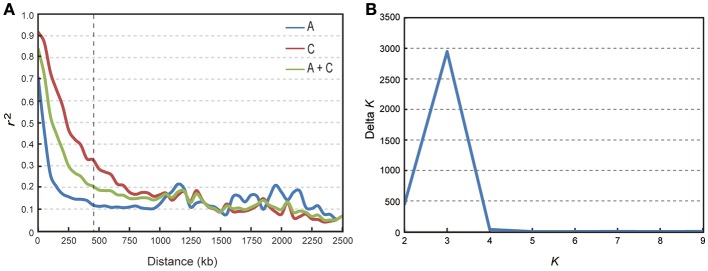
Analysis of LD decay and the population structure of 333 rapeseed accessions. **(A)** Subgenome and genome distribution of *r*^2^-value (linkage disequilibrium decay, LD) estimated from 333 rapeseed accessions. **(B)** Calculation of Δ *K* based on the value of Ln P(D) between successive *K*-values.

We calculated LD in 333 accessions using the parameter *r*^2^ with the 34,292 SNP markers. In this analysis, three chromosomes (A08, C02, and C07) showed strong LD, with distances ranging from 1,300 to 1,710 kb; while others exhibited modest LD, with distances ranging from 100 to 825 kb when *r*^2^ = 0.2 (Table 2). The average LD decay of chromosomes A and C were of 190 and 700 kb respectively, while the average LD decay of the whole genome (A + C chromosomes) was of 450 kb when *r*^2^ = 0.2 (Sun F. M. et al., 2016; Figure 2A).

### Genome-wide association study

**Figure 3:**
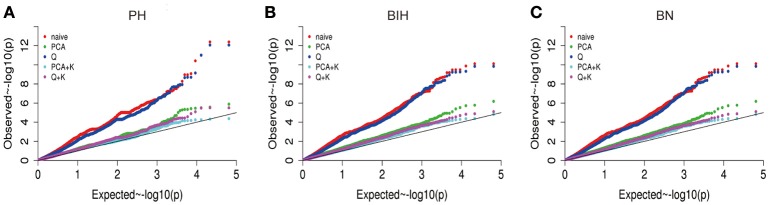
Quantile-quantile plots of estimated −log_10_ (*p*-value) for PH **(A)**, BIH **(B)**, and BN **(C)** using five models. PH, plant height; BIH, branch initation height; BN, branch number.

**Figure 4:**
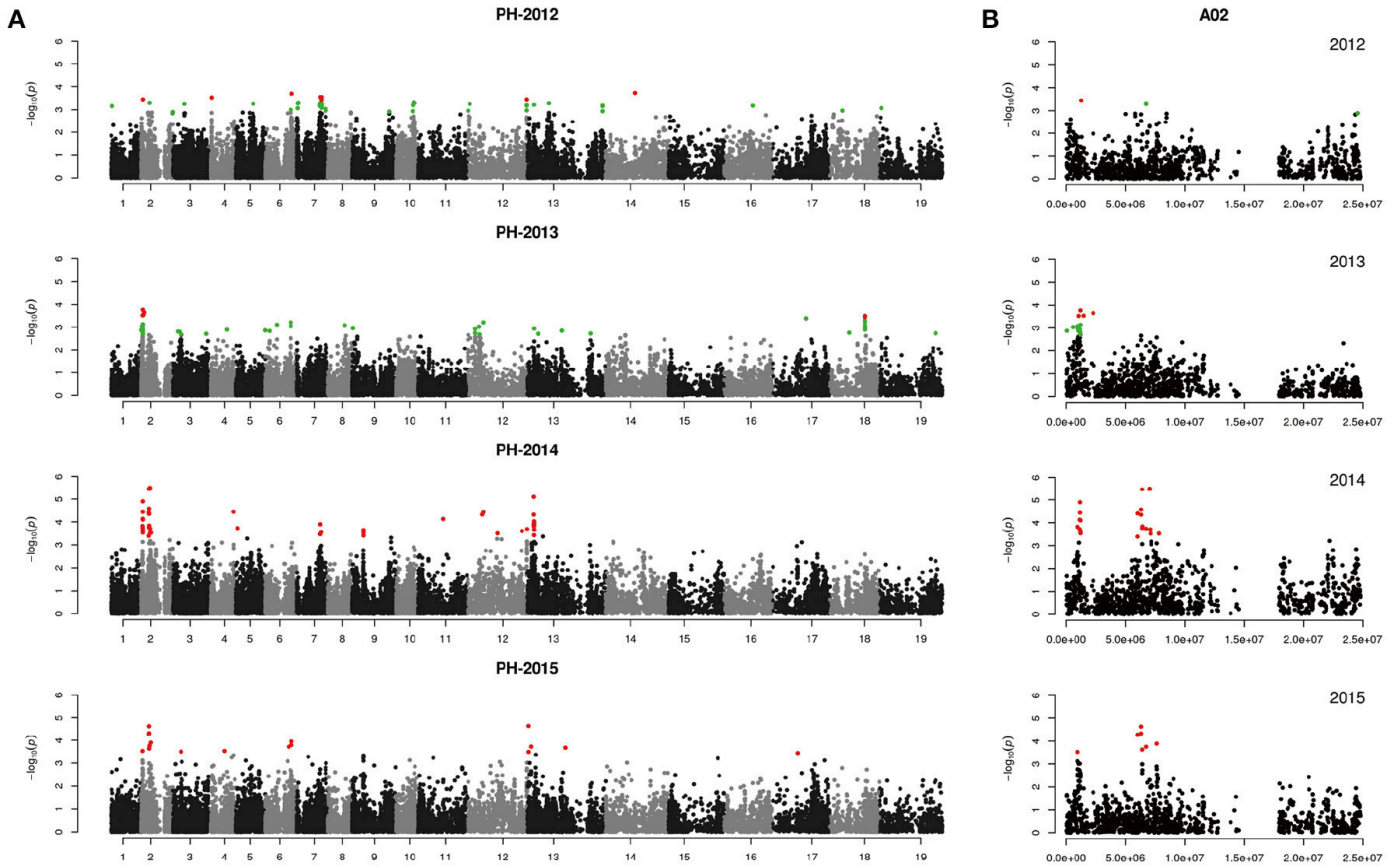
Genome-wide association study of plant height in the panel of 333 accessions. **(A)** Manhattan plot of the MLM for plant height in 4 years. **(B)** Main locus on chromosome A02 for plant height regulation. Red color plots are identified by the Q+K model, green color plots are identified by the top 50 method.

**Figure 5:**
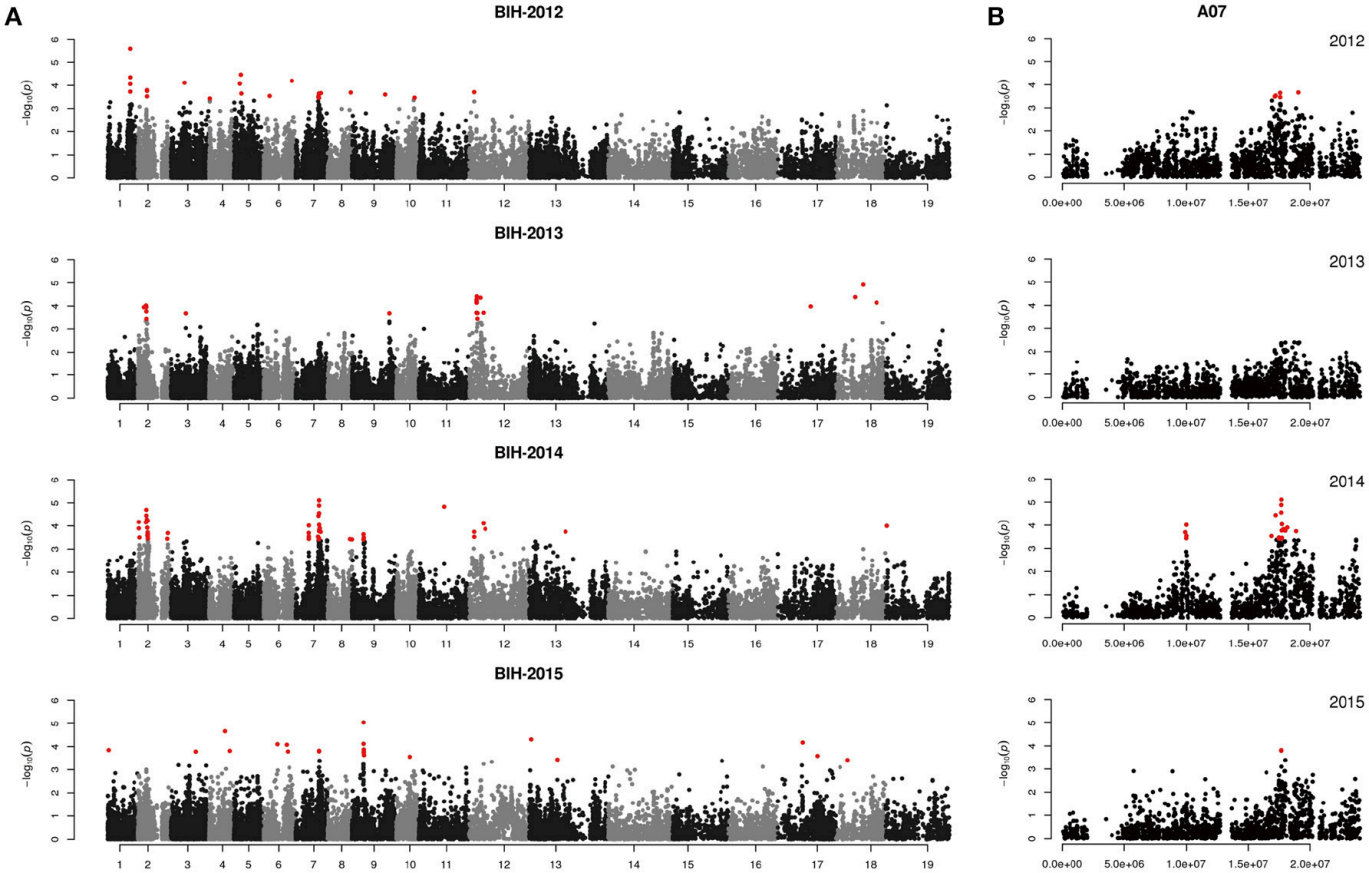
Genome-wide association study of branch initiation height in the panel of 333 accessions. **(A)** Manhattan plot of the MLM for branch initiation height in 4 years. **(B)** Main locus on chromosome A07 for branch initiation height regulation.

**Figure 6:**
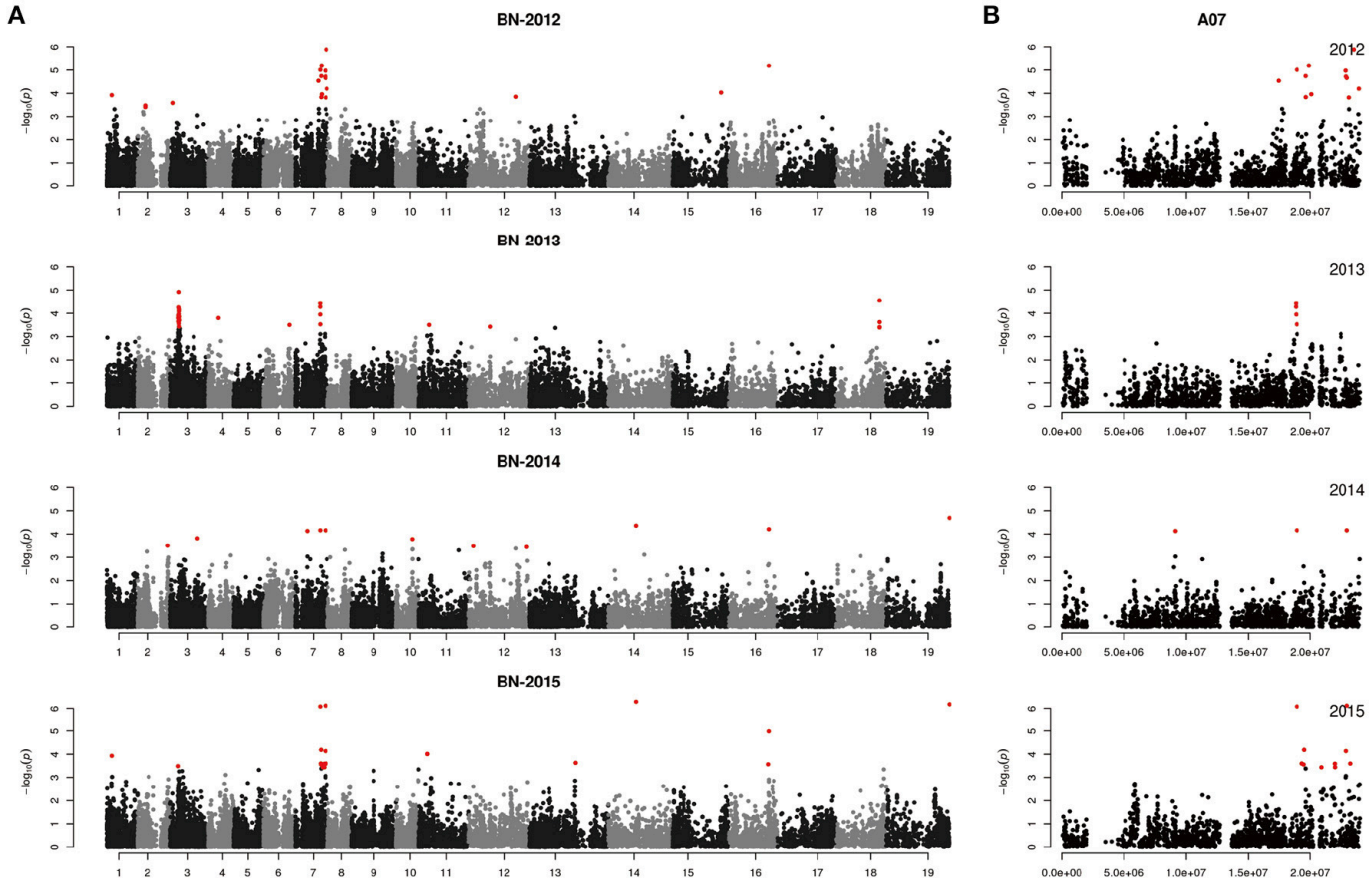
Genome-wide association study of branch number in the panel of 333 accessions. **(A)** Manhattan plot of the MLM for branch number in 4 years. **(B)** Main locus on chromosome A07 for branch number regulation.

We performed GWAS with mixed linear modeling (MLM) across 4 years. For the groups-populations, we created a Q matrix (*k* = 2) for all 333 accessions, in order to obtain the highest Δ *k*-value (Figure 2B). In addition, minor differences were observed among the PCA, Q, K, Q + K, and PCA + K models, and the Q + K model is an appropriate model (Figure 3). Thus, the Q + K model was used for GWAS. As in other GWAS studies, we set a threshold of −log_10_ *P* = 3.39 as a significant association (Figure S1; Benjamini and Hochberg, 1995; Wang et al., 2016). Furthermore, QQ plots of PH in 2012 and 2013 were not linear. As a result, the SNPs showing the 50 highest −log_10_ *P*-values in each year were compiled for the PH trait for these 2 years to identify potentially significant SNP clusters (Figure 4; Ueda et al., 2015). Overall, for all traits analyzed, a total of 158 SNPs associated with PH, BIH, and BN were identified (Table S2). Furthermore, LD blocks (450 kb) harboring the repeating SNPs across the 4 years (and detected in at least two of those years) were identified as regions containing putative candidate loci. This led to the selection of 38 SNPs (Table 3), which in turn could be merged into seven, four, and five loci for PH, BIH, and BN, respectively (Figures 4–**6**, Table 3, Figures S2–S4).

**Table 3:** Genome-wide significant associations for PH, BIH, and BN identified by the MLM.

**Trait**	**Chr**	**Range (bp)**	**Marker**	**Pos**	**Environment**	**Major allele**	**Minor allele**	**Minor allele frequenc**.	***P***	**Max [−log_10_(*P*)]**	**Contribution (%)**	
PH	A02	575,355–1,481,113	Bn-A02-p3530703	965813	2014; 2015	G	A	0.35	0.0001522	3.82	6.98	QTL in 3/4 year
			Bn-A02-p3735101	1160348	2013; 2014	A	G	0.2	7.424E-05	4.13	7.35	
			Bn-A02-p3759462	1181964	2013; 2014	G	A	0.17	3.656E-05	4.44	7.84	
			Bn-A02-p3760177	1182739	2013; 2014	G	A	0.16	1.27E-05	4.90	8.70	
			Bn-A02-p3796695	1219994	2013; 2014	G	A	0.15	7.93E-05	4.10	7.33	
			Bn-A02-p3803198	1223532	2013; 2014	G	A	0.17	0.0002916	3.54	6.20	
	A02	6,034,589–7,113,740	Bn-A02-p9240182	6043399	2014; 2015	A	C	0.08	3.929E-05	4.41	7.77	
			Bn-A02-p9505646	6319840	2014; 2015	G	A	0.09	2.35E-05	4.63	9.79	
			Bn-A02-p9506103	6320247	2014; 2015	G	A	0.1	4.493E-05	4.35	8.90	
			Bn-A02-p9607756	6411279	2014; 2015	A	G	0.41	3.36E-06	5.47	9.75	
			Bn-A02-p9888208	6733048	2012; 2014; 2015	G	A	0.07	0.0001756	3.76	5.62	
	A06	19,756,036–20,526,564	Bn-A06-p18369013	19740118	2012; 2013	G	A	0.34	0.0008984	3.05	5.16	
	C03	4,638,640–5,080,194	Bn-scaff_28429_1-p467447	4908423	2012; 2014	A	G	0.41	9.50E-05	4.02	5.80	
			Bn-scaff_21778_1-p160881	4962469	2013; 2014	C	A	0.22	1.45E-04	3.84	5.65	
	A07	17,918,961–19,318,942	Bn-A07-p16015935	17918961	2012;2014	A	G	0.14	0.0003404	3.47	6.12	QTL in 2 year
			Bn-A07-p16020955	17923677	2012; 2014	A	C	0.14	0.0003402	3.47	6.11	
			Bn-A07-p16039345	17954858	2012; 2014	C	A	0.14	0.0003402	3.47	6.11	
			Bn-A07-p16050076	17964471	2012; 2014	C	A	0.14	0.0003402	3.47	6.11	
	A03	6,196,436–6,245,297	Bn-A03-p6909237	6196436	2015	A	C	0.45	3.17E-04	3.50	7.25	
			Bn-A03-p6967307	6245297	2013	G	A	0.3	0.00204	2.69	3.48	
	C02	45,551,420–45,848,554	Bn-scaff_16139_1-p921208	45588698	2012	G	A	0.26	0.000613	3.21	6.00	
			Bn-scaff_16139_1-p662939	45848554	2014	G	A	0.17	2.08E-04	3.68	7.67	
BIH	A02	6,043,399–7,494,669	Bn-A02-p9240182	6043399	2013; 2014	A	C	0.08	6.99E-05	4.16	7.32	QTL in 3/4 year
			Bn-A02-p9505646	6319840	2013; 2014	G	A	0.09	2.01E-05	4.70	9.36	
			Bn-A02-p9506103	6320247	2013; 2014	G	A	0.1	3.53E-05	4.45	9.26	
	A07	16,890,645–18,144,511	Bn-A07-p15303340	17219698	2012; 2014	C	A	0.23	3.63E-05	4.44	8.01	
			Bn-A07-p15756045	17662651	2014; 2015	A	G	0.28	2.80E-05	4.55	8.08	
			Bn-A07-p15758978	17665715	2014; 2015	C	A	0.27	1.31E-05	4.88	8.73	
	A09	8,736,879–9,093,352	Bn-A09-p9669847	8736879	2014; 2015	C	A	0.46	9.07E-06	5.04	9.87	QTL in 2 year
			Bn-A09-p9671359	8740105	2014; 2015	G	A	0.24	7.58E-05	4.12	7.63	
			Bn-A09-p9877560	8899345	2014; 2015	G	A	0.23	1.36E-04	3.87	7.15	
			Bn-A09-p9878665	8900449	2014; 2015	A	G	0.23	1.36E-04	3.87	7.15	
	A08	18,031,286–18,912,280	Bn-A08-p20551301	18031286	2012	G	A	0.07	2.04E-04	3.69	5.91	
			Bn-A08-p20801363	18912280	2014	A	G	0.32	3.84E-04	3.42	6.12	
BN	A07	18,835,388–23,926,969	Bn-A07-p16996070	18916365	2012; 2014; 2015	A	C	0.17	8.26E-07	6.08	11.52	QTL in 3/4 year
			Bn-A07-p21344843	22897178	2012; 2015	A	C	0.24	1.89E-05	4.72	7.68	
			Bn-A07-p21413042	22963184	2012; 2014; 2015	A	G	0.16	7.63E-07	6.12	11.54	
	A01	3,733,914	Bn-A01-p4070059	3733914	2012; 2015	A	G	0.26	1.17E-04	3.93	7.46	QTL in 2 year
	C04	20,824,102	Bn-scaff_20567_1-p64644	20824102	2014; 2015	G	A	0.1	5.15E-07	6.29	12.17	
	C09	48,401,790	Bn-scaff_28053_1-p60542	48401790	2014; 2015	A	G	0.06	6.62E-07	6.18	12.70	
	A03	6,245,297–6,972,809	Bn-A03-p7326202	6605382	2013	G	A	0.42	1.20E-05	4.92	8.61	

For PH, we detected nine GWAS peak SNPs in the seven loci located on chromosomes A02, A06, C03, and A07, and five of which were detected in at least 2 years (Figure 4, Table 3). These seven peak SNPs explained 35.56% of the total phenotypic variance, with the largest contribution (*R*^2^ ~9.79%) from SNP Bn-A02-p9505646 (Table 3). For BIH, four peak SNPs were detected on chromosomes A02, A07, A08, and A09, and three of these SNPs were detected in at least 2 years (Figure 5, Table 3). These three SNPs explained 27.96% of the phenotypic variance (Table 3). SNP Bn-A02-p9505646 was detected in both PH and BIH traits and it accounted for 9.36% of the total phenotypic variance in BIH (Table 3). For BN, five peak SNPs were detected on chromosomes A01, A03, A07, C04, and C09 (Figure 6), two of which were detected in at least 3 years, with the largest contribution (*R*^2^ ~11.54%) from SNP Bn-A07-p21413042 (Table 3).

### Allele-specific SNP markers correlated with physical traits (PH, BIH, and BN)

**Figure 7:**
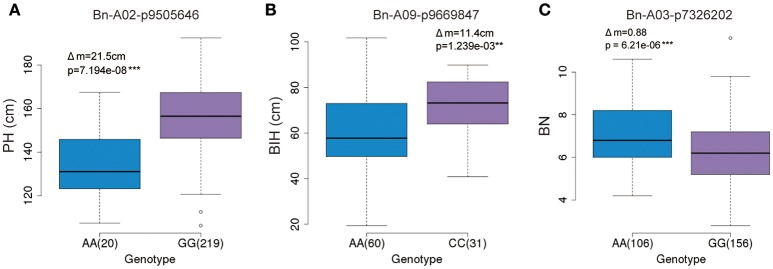
Allelic effects of associated SNPs for three traits. **(A)** Phenotypic difference between AA and GG with marker Bn-A02-p9505646 for plant height in the panel. **(B)** Phenotypic difference between AA and CC with marker Bn-A09-p9669847 for branch initiation height in the panel. **(C)** Phenotypic difference between AA and GG with marker Bn-A03-p7326202 for branch number in the panel.

The GWAS identified several significant SNP markers in the regulation of three traits (Table 3). To find useful SNP markers for marker-based breeding, we estimated the allelic effect of the peak SNPs across these three traits. The marker Bn-A02-p9505646 (G/A) of the GG allele showed the largest contribution (*R*^2^ ~9.79%) to PH regulation, measuring 21.5 cm more than those of the AA allele (*P* < 0.05; Figure 7A). Apart from the Bn-A02-p9505646 marker, individuals with the CC allele marker Bn-A09-p9669847 showed the largest contribution to BIH regulation (*R*^2^ ~9.87%) and measured 11.4 cm more than those of the AA allele (*P* < 0.05; Figure 7B). The average BN of individuals with the AA allele with marker Bn-A03-p7326202 showed the higher contribution (*R*^2^~8.61%) and was significantly greater than GG alleles on chromosome A3 (Figure 7C). These SNP markers may permit marker-based breeding for rapeseed plant architecture.

### Identification of candidate genes

We searched for candidate genes from the genomic regions with significant SNPs according to GO annotations from the Arabidopsis database (http://www.arabidopsis.org/index.jsp). Notably, 31, 15, and 17 candidate genes for PH, BIH, and BN were located in each region, respectively (Table S3).

For PH, the main QTL was detected on chromosome A02, located at 6.0~6.7 M. From the peak SNP Bn-A02-p9607756 [−log_10_ (*p*) = 5.47], two candidate genes associated with GA signaling and flowering time repression were located at 77.2 and 29.4 kb, respectively (Table 4). They encode proteins GAI-DELLA (BnaA02g12260D) and FT (BnaA02g12130D), which may be involved in PH formation (Wang and Li, 2008; Salas Fernandez et al., 2009). A BR biosynthesis gene CYP450 (BnaA02g02150D), a gibberellin-regulated protein GASA4 (BnaA02g02560D), and two cell wall biosynthesis proteins (BnaA02g02700D and BnaA02g03310D) were identified up- and down-stream of the peak SNP of Bn-A02-p3760177 [−log_10_ (*p*) = 4.90] (Table S3). In addition, we also identified four candidate genes for PH regulation on chromosomes A06 and A07 (Table 4).

**Table 4:** Putative candidate genes for PH, BIH, and BN.

**Trait**	**Gene ID**	**Chr**	**Pos. star**	**End**	**Annotation**	**Function**
PH	BnaA02g12130D	A02	6375936	6379058	FT-like	PEBP family protein, promotes flowering
	BnaA02g12260D	A02	6485638	6486225	RGA	GRAS family transcription factor family protein, involved in gibberellic acid mediated signaling
	BnaA06g30410D	A06	20546076	20548307	RGA	GRAS family transcription factor, act early in the phytochrome A signaling pathway
	BnaA06g30720D	A06	20745499	20748444	BR6ox2	Encodes a cytochrome p450 enzyme that catalyzes the last reaction in the production of brassinolide
	BnaA07g25310D	A07	18855196	18857952	FT-like	PEBP family protein, together with LFY, promotes flowering
	BnaA07g25390D	A07	18893860	18898414	ARF8	Mediates auxin response via expression of auxin regulated genes
BIH	BnaA02g13870D	A02	7665493	7667213	C2H2 finger	Induce the proliferation of lateral organ tissue
	BnaA02g14010D	A02	7832018	7833120	TCP12	Arrest axillary bud development and prevents axillary bud outgrowth
	BnaA02g14060D	A02	7901790	7902712	SOB five-like 2	Positive regulator of cytokinin levels and cytokinin-mediated development
	BnaA07g24210D	A07	18082422	18082784	CLAVATA3	Encoding small peptides with conserved carboxyl termini
	BnaA07g24240D	A07	18123092	18124164	LOF2	MYB-domain transcription factor, functions in boundary specification and meristem initiation
	BnaA09g14730D	A09	8538776	8540793	MATE efflux family protein	Encodes a plant MATE (multidrug and toxic compound extrusion) transporter that is involved in determining the rate of organ initiation.
BN	BnaA07g24890D	A07	18648077	18650015	ARR11	Acts in concert with other type-B ARRs in the cytokinin signaling pathway
	BnaA07g25110D	A07	18761213	18763618	TCP12	Encodes protein with TCP (TB1,CYC,PCF) domain
	BnaA07g25390D	A07	18893860	18898414	ARF8	Mediates auxin response via expression of auxin regulated genes
	BnaA07g28100D	chrA07	20329509	20330648	LOF2	MYB-domain transcription factor, functions in boundary specification and meristem initiation
	BnaA07g28210D	A07	20382967	20384750	CUC3	NAC (No Apical Meristem) domain transcriptional regulator
	BnaA01g08170D	A01	3871348	3873138	YUCC8	Flavin-binding monooxygenase family protein, IAA biosynthesis
	BnaA01g08280D	A01	3942360	3944177	IAA11	Auxin induced gene, acts in auxin-activated signaling pathway
	BnaC09g49730D	C09	47953828	47954525	WOX5	WUSCHEL related homeobox 5

For BIH, the C2H2 zinc finger protein, TCP 12, and SOB five-like 2 (BnaA02g13870D, BnaA02g14010D, and BnaA02g14060D) were also identified in the candidate region around the peak SNP Bn-A02-p9505646 [−log_10_ (*p*) = 4.70] on chromosome A02 (Table 4). On chromosome A07, a CLAVATA3 protein and a LOF2 transcription factor (BnaA07g24210D and BnaA07g24240D) were identified downstream from the peak SNP Bn-A07-p15758978) [−log_10_ (*p*) = 4.88] (Table 4). Their homologs in Arabidopsis are also involved in meristem initiation and maintenance (Lee et al., 2009). In addition, on chromosome A09, we found a peak SNP Bn-A09-p9669847 with the highest probability of marker-trait association, with −log_10_ (*p*) = 5.04, and with the largest contribution to BIH variation, with *R*^2^ = 9.87%. Notably, a MATE efflux family protein (BnaA09g14730D) involved in determining the rate of organ initiation was identified close to the peak SNP (Table 4).

For BN, most candidate genes around peak SNPs were associated with Auxin, CK, and SL (Table S2). Five candidate genes, *ARR11, TCP12, ARF8, LOF2*, and *CUC3*, were identified up- and downstream of the peak SNP Bn-A07-p16996070 [−log_10_ (*p*) = 6.08] (Table 4). Another peak SNP Bn-A01-p4070059 [−log_10_ (*p*) = 3.93] contained two candidate genes (*YUCC8* and *IAA11*). On chromosome C09, a *WOX*5 gene (BnaC09g49730D) was located 0.45 M from the peak SNP Bn-scaff_28053_1-p60542 [−log_10_ (*p*) = 6.18] (Table 4).

## Discussion

In our study, a MLM was used to calculate the association in the GWAS analysis, while incorporating a Q matrix and kinship data to control the false discovery rate, this model was an improvement relative to the naïve GLM (Figure 3; Larsson et al., 2013). Although it has been suggested that PCA or PCA + K models may reduce false positives in GWAS analyses (Yu et al., 2006; Zhu et al., 2008; Li et al., 2016), many studies have used the Q + K model because it permits control of the false discovery rate (Cai et al., 2014; Sun F. M. et al., 2016; Wang et al., 2016). In our assays, even the QQ plots of PH deviated from the expected distribution, while the Manhattan plots showed clearly defined peaks, indicating that MLM might be the best approach to detect the main QTLs for our traits (Figures 4–6, Figure S1). According to previous studies, we defined the SNPs which less than the *p*-value of 4.06 × 10^−4^ [−log_10_ (*p*-value) = 3.39, Q + K] or in the top 50 SNPs in PH control as the candidate SNPs (Ueda et al., 2015; Wang et al., 2016). The LD decay is the key factor for mining candidate genes in a GWAS study (Yu and Buckler, 2006). We observed that LD decay in the A- and C-subgenomes is different in our panel. The LD decay in the A- subgenome was generally shorter than those in the C- subgenome, while the LD decays on A08, C02, and C07 were beyond 1.3 M. Our results for LD decay rates are similar to those found in recent GWAS studies on rapeseed (Qian et al., 2014; Liu et al., 2016), and therefore, we hold that the LD decay in our study is reliable to identify candidate genes.

### Novel genetic control of three plant architecture traits (PH, BIH, and BN) in rapeseed

GWAS was used to detect QTLs of PH, BIH, and BN, which are main factors regulating plant architecture in rapeseed. According to the marker-trait association SNPs, we identified seven QTLs for PH, four for BIH, and five for BN (Table 3). In the PH candidate loci, these QTLs were distributed on six chromosomes and some QTL regions were similar to those identified in previous studies. Among them, the QTLs at 1.2 and 6.4 M on chromosome A02 overlapped regions detected by Udall et al. (2006); Li X. N. et al. (2013), and Sun et al. (2016b). The QTL at 18.0 M on chromosome A07 was close to the QTLs detected by Udall et al. (2006) and Li et al. (2016). The QTL near the top of chromosome C03 was also detected by Ding et al. (2012) and Shi et al. (2009). Additionally, the QTL at 6.2 M on chromosome A03 was found by Mei et al. (2009) and Li X. N. et al. (2013), and also corresponds closely to the findings of Li et al. (2016). The QTL at 45.8 M on chromosome C02 corresponds closely to the QTL on the bottom of C02 detected by Udall et al. (2006). These results indicate that these QTLs were stable as they were detected by different map populations and in different environments using distinct methods of analysis, and they may also have been selected in rapeseed breeding. Notably, the QTL at 19.7 M of A06, which was detected in 2 years has not been found in previous studies, and may be a novel QTL.

For the BN candidate loci, only one QTL was located at 6.6 M on chromosome A03, as shown previously in other studies (Shi et al., 2011; Li et al., 2016). Interestingly, we found that all the other four QTLs were detected in a rapeseed accession that has a stable BN but increased branching. In our study, most of the accessions are varieties cultivated from different areas, we therefore deduced that the four QTLs might have converged or have been artificially selected in rapeseed breeding. However, several QTLs of PH and BN regulation reported in other studies were not detected in this study. This may be ascribed to insufficient numbers of rapeseed accessions in GWAS or to diversity of climate. In addition, all QTLs might not have been detected due to an insufficient density of SNPs in some genomic regions.

Previously, only one study identified QTLs associated with the height of the lowest primary effective branch (HPB) using two populations in rapeseed, and it identified 10 QTLs distributed on chromosomes A02, A07, A08, C04, C06, and C07 (Chen et al., 2007). In our study, BIH was measured from the ground level up to the base of the lowest primary effective branch, and four QTLs were detected in our natural population. One of these QTLs was located at 18.0 M on chromosome A08, which corresponds to the QTL detected on the bottom of the same chromosome by Chen et al. (2007). Two more QTLs were found on corresponding chromosomes A02 and A07. However, their specific regions did not overlap. To verify the reliability of these QTLs, further investigations of this trait must be undertaken.

In our study, the QTLs at 6.3 M on chromosome A02 and at 18.0 M on chromosome A07 influenced PH and BIH, and a significant correlation was found between these two traits during our correlation assay. In the A02 QTL regions, PH and BIH traits shared three SNPs and the two traits were also co-localized, which suggests pleiotropic regulation of a single gene or the existence of closely linked genes. In contrast, in the A07 QTLs, the SNP associated with the PH QTL was separated from the related SNP associated with the BIH QTL by about 700 kb, while the LD (*r*^2^) between the two SNPs was 0.27, indicating that they are separate QTLs. In addition, QTLs related to PH on chromosome A03 and A07 were found close to the QTLs related to BN on the same chromosomes, at a distance of 500 kb and 1 M, respectively. The QTL at 17.7 M on chromosome A07 related to BIH regulation was close to the QTL related to BN, at ~1.3 M. These distances are also beyond the average LD decay and the LD (*r*^2^) between the two peak SNPs for each group were relatively low, suggesting that they are separate QTLs.

### Candidate genes for PH and BN regulation

In Arabidopsis, rice, and other plant species, BR, IAA, GA, and SL biosynthesis and signaling pathways are known to regulate PH, while mutations in most of these genes cause dwarf phenotypes (Wang and Li, 2008; Sun et al., 2010; Clouse, 2011). Additionally, genes involved in flowering time also play a role in PH regulation, such as DTH8 and Ghd7 in rice (Xue et al., 2008; Wei et al., 2010), and Flt-2L in wheat (Chen et al., 2009). In rapeseed, Mei et al. (2009) found an overlap between a flowering time locus and PH QTLs. Moreover, in some genes regulating cell wall formation, like cellulose synthase genes, mutations affecting cell elongation lead to dwarf phenotypes (Tanaka et al., 2003). Therefore, we defined PH regulation candidate genes in rapeseed based on similarity to orthologs of the QTL regions. Using the average LD decay, 5 genes (16.7%) are related to BR biosynthesis or signaling pathways, 6 genes (20%) are related to GA, 2 genes (6.7%) are associated with Auxin, 4 genes (13.3%) are involved in cell wall formation, 4 genes (13.3%) are related to flowering time regulation, and 2 gene (6.7%) are related to trichome branching development in Arabidopsis (Bischoff et al., 2010; Table S3). Notably, the *RGA* gene, encoding a DELLA protein, displays a signal close to Bn-A02-p9505646, which itself is close to the Bn-A02-p9610453 marker identified in Sun et al. (2016b). Furthermore, we found that some flowering time QTLs (*BnFT*) are also located in the same region of chromosome A02 (Xu et al., 2016). In Arabidopsis and rice, most studies showed that *FLOWERING LOCUS T* (*FT*) and the rice *FT* homolog *HEADINGDATE3a (Hd3a)* gene affected flowering and PH (Kardailsky et al., 1999; Kobayashi et al., 1999; Tamaki et al., 2007). In addition, overexpressed the FT in tobacco also exhibited the dwarf phenotype (Lewis and Kernodle, 2009). Thus, we deduce that *BnRGA* (*BnaA02g12260D*) and *BnFT* (*BnaA02g12130D*) are main candidate genes on chromosome A02 that are involved in PH regulation.

Several transcription factors (TF) and hormones (Auxin, CKs, and SLs) also affect shoot outgrowth in plant architecture (Yang and Jiao, 2016). We compared these orthologs to our main candidate genes for BN. Four genes (23.5%) are related to CK biosynthesis or signaling pathways, 6 genes (35.3%) are related to IAA, 3 genes (17.6%) are associated with flowering or flower identity formation, and one gene is involved in the SL signaling pathway. Many recent studies report that SL is functionally important in BN and PH regulation; however, only one candidate gene was identified in our GWAS. This may be due to a low density of SNP markers, or due to the candidate genes being farther away from the QTL locus than the average LD decay, or due to the confounding of the population structure. In Arabidopsis, *LATERAL ORGAN FUSION2* (*LOF2*) encodes a MYB-domain transcription factor that functions in both lateral organ separation and axillary meristem formation, in part through interaction with *CUC2, CUC3*, and *STM* (Raman et al., 2008; Lee et al., 2009). In novel loci on chromosome A07, genes *BnLOF2* (BnaA07g26170D) and *BnCUC3* (BnaA07g28210D) were identified as the most likely candidate genes and may play roles in both organ separation and axillary meristem formation.

Although several candidate genes were identified following the GWAS, the function of these genes remains ambiguous. In the future, it is necessary to isolate target genes by cloning these putative genes or by developing mapping populations for the QTLs, and to illustrate their functional role in relative trait regulation by transformation experiments.

## Author contributions

MZ, WH, and HW conceived and designed the research; HL performed GWAS; XW, MT, HY, XL, JL, and XS characterized the agronomic traits; MZ, CP, HL, and JX analyzed the data; and MZ wrote the manuscript. All authors read and approved the final manuscript.

### Conflict of interest statement

The authors declare that the research was conducted in the absence of any commercial or financial relationships that could be construed as a potential conflict of interest.

## Supplementary material

Figure S1 Quantile-quantile plots of the estimated –log_10_ (*p*-value) for PH, BIH, and BN, using Q+K models.

Figure S2 Close-up of the other five loci on chromosomes for PH regulation.

Figure S3 Close-up of the other three loci on chromosomes for BIH regulation.

Figure S4 Close-up of the other four loci on chromosomes for BN regulation.

Table S1 Correlation analysis among the three traits.

Table S2 Significant-SNP for PH, BIH, and BN identified by the MLM.

Table S3 Putative candidate genes for PH, BIH, and BN.
