# Supplementary figures and images for: Genome-Wide Association Study Reveals Candidate Genes for Control of Plant Height, Branch Initiation Height and Branch Number in Rapeseed (Brassica napus L.)
Source: Front Plant Sci. 2017 Jul 18;8:1246. doi: 10.3389/fpls.2017.01246 (PMC5513965; doi:10.3389/fpls.2017.01246)

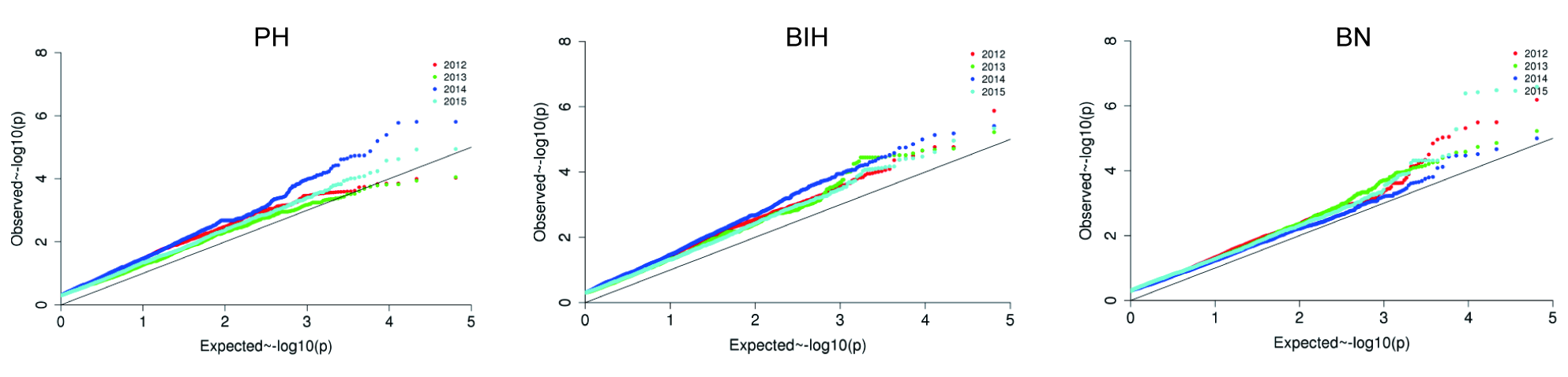

Supplement: Figure S1 — Quantile-quantile plots of the estimated –log10 (p-value) for PH, BIH, and BN, using Q+K models. [file Image1.TIF]

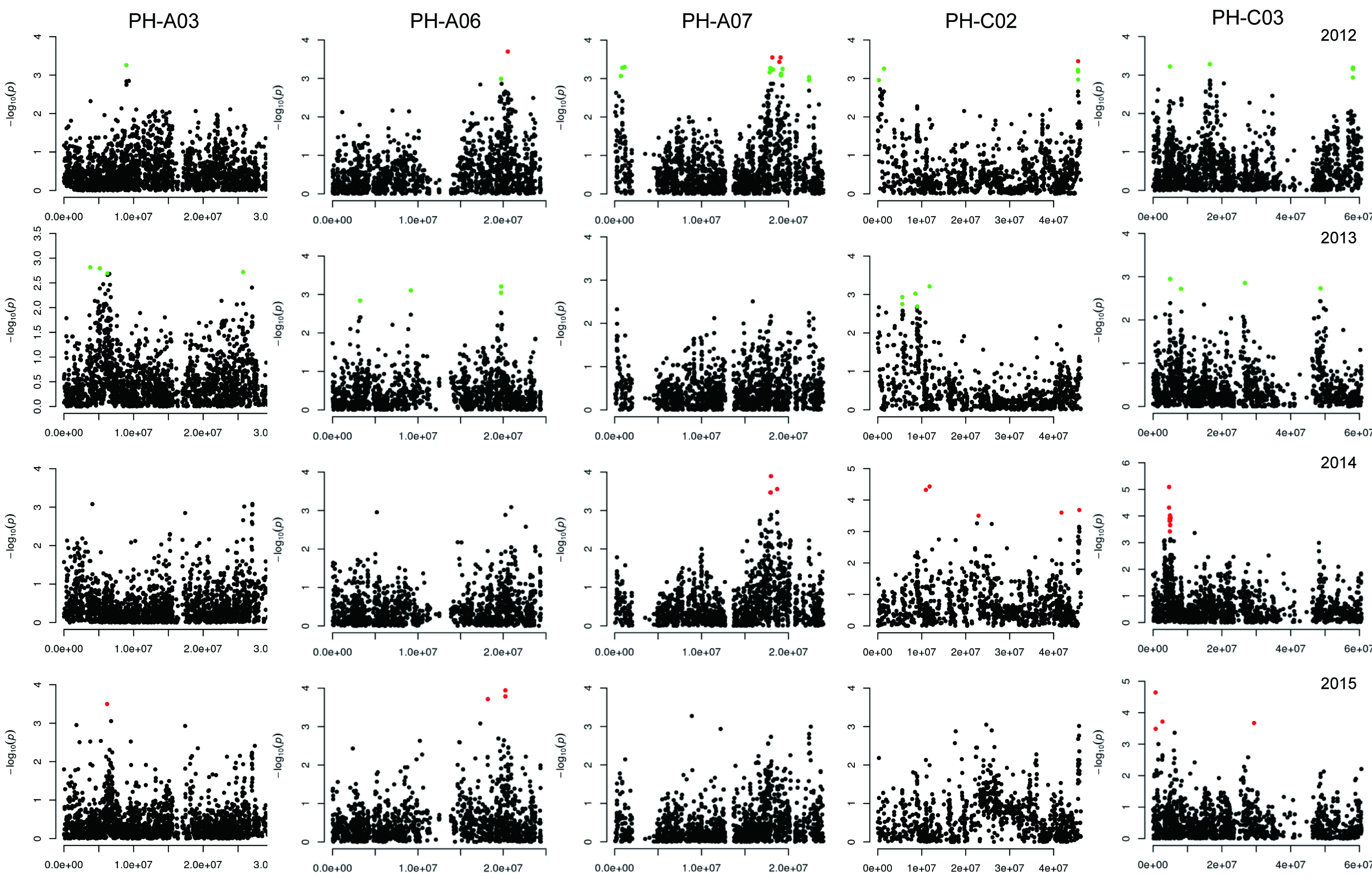

Supplement: Figure S2 — Close-up of the other five loci on chromosomes for PH regulation. [file Image2.TIF]

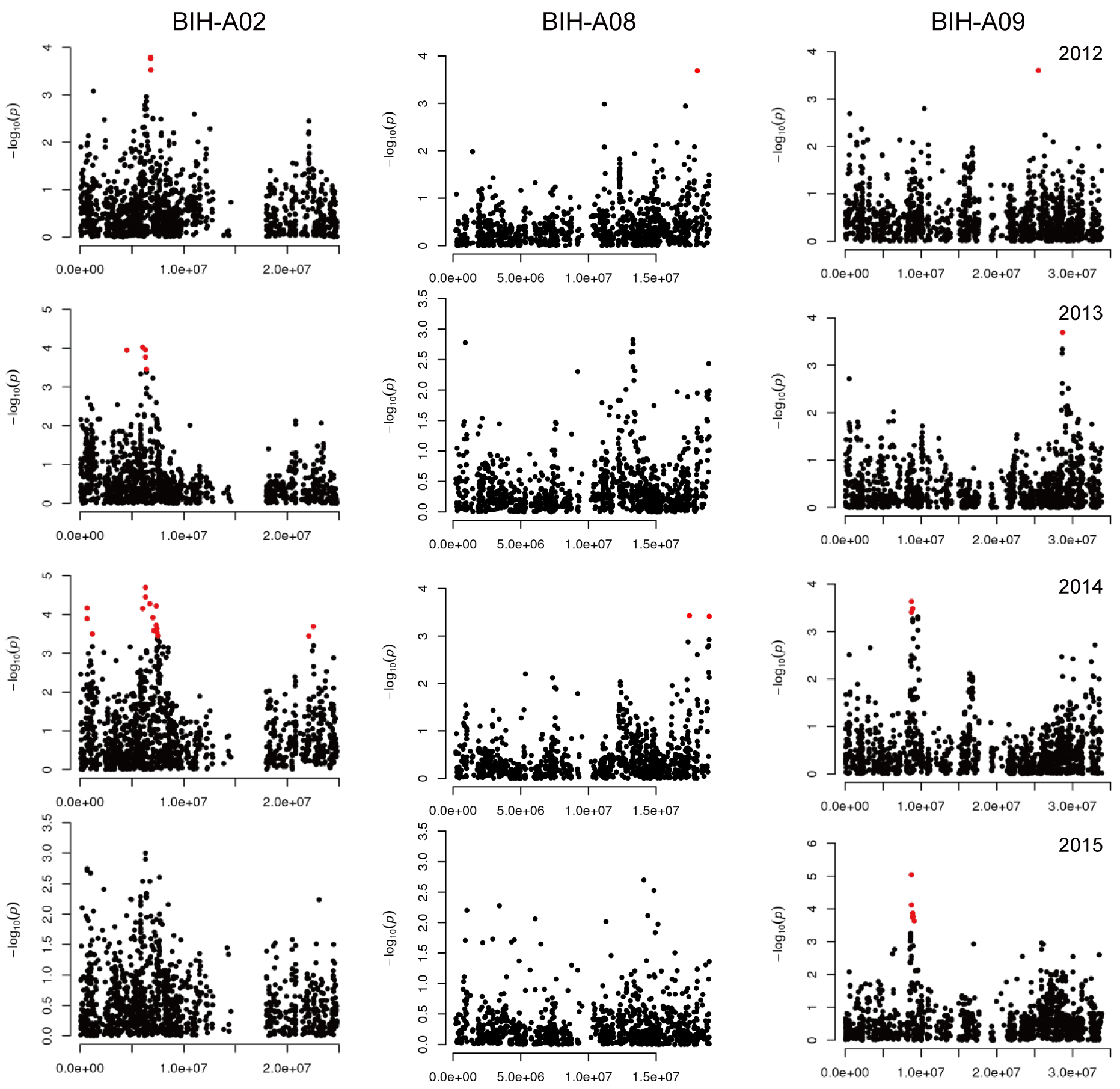

Supplement: Figure S3 — Close-up of the other three loci on chromosomes for BIH regulation. [file Image3.TIF]

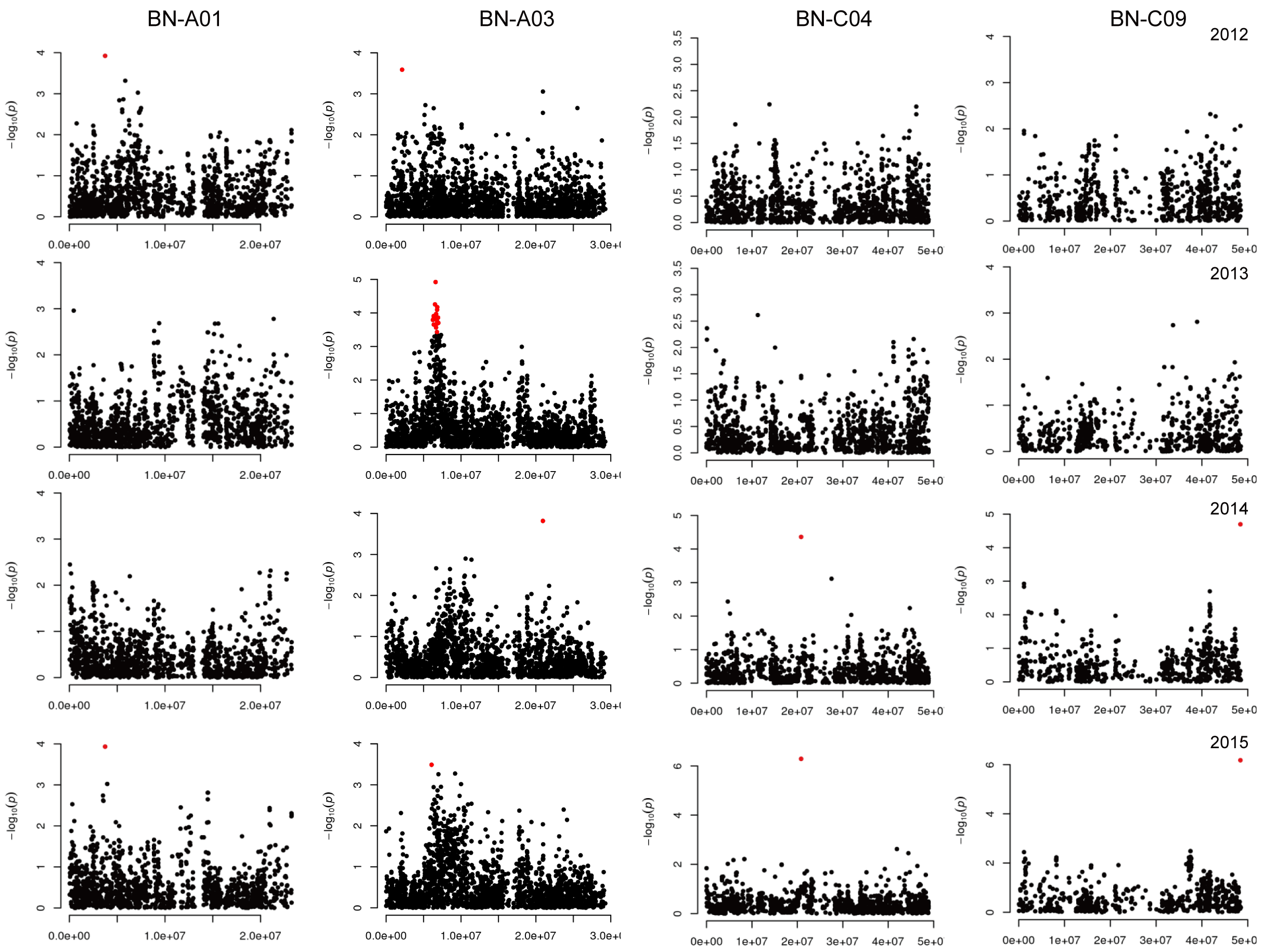

Supplement: Figure S4 — Close-up of the other four loci on chromosomes for BN regulation. [file Image4.TIF]
