# Supplementary material for: Genome-Wide Association Study Reveals Candidate Genes for Control of Plant Height, Branch Initiation Height and Branch Number in Rapeseed (Brassica napus L.)
Source: Front Plant Sci. 2017 Jul 18;8:1246. doi: 10.3389/fpls.2017.01246 (PMC5513965; doi:10.3389/fpls.2017.01246)
Supplement: Table S1 — Correlation analysis among the three traits. [file Table1.DOCX]

**Table S1** Correlation analysis among the three traits.

|  |  | **BIH1** | **BIH2** | **BIH3** | **BIH4** | **BN1** | **BN2** | **BN3** | **BN4** |
| --- | --- | --- | --- | --- | --- | --- | --- | --- | --- |
| **PH1** | Pearson’s correlation coefficient | .500** | .377** | .436** | .498** | .316** | .160* | .183** | 0.029 |
|  | P value | 0 | 0 | 0 | 0 | 0 | 0.019 | 0.007 | 0.674 |
| **PH2** | Pearson’s correlation coefficient | .324** | .623** | .435** | .472** | 0.016 | .433** | 0.029 | 0.076 |
|  | P value | 0 | 0 | 0 | 0 | 0.819 | 0 | 0.673 | 0.27 |
| **PH3** | Pearson’s correlation coefficient | .397** | .434** | .734** | .607** | -0.014 | 0.097 | 0.128 | -0.018 |
|  | P value | 0 | 0 | 0 | 0 | 0.839 | 0.157 | 0.061 | 0.788 |
| **PH4** | Pearson’s correlation coefficient | .431** | .515** | .624** | .763** | -0.014 | .206** | 0.112 | .146* |
|  | P value | 0 | 0 | 0 | 0 | 0.84 | 0.003 | 0.102 | 0.032 |
| **BN1** | Pearson’s correlation coefficient | -.306** | -0.08 | -.169* | -0.056 |  |  |  |  |
|  | P value | 0 | 0.245 | 0.013 | 0.414 |  |  |  |  |
| **BN2** | Pearson’s correlation coefficient | -0.017 | 0.041 | 0.029 | 0.078 |  |  |  |  |
|  | P value | 0.8 | 0.548 | 0.678 | 0.258 |  |  |  |  |
| **BN3** | Pearson’s correlation coefficient | -0.115 | -0.073 | -.235** | -0.095 |  |  |  |  |
|  | P value | 0.094 | 0.287 | 0.001 | 0.168 |  |  |  |  |
| **BN4** | Pearson’s correlation coefficient | -.140* | -0.097 | -.217** | -.208** |  |  |  |  |
|  | P value | 0.04 | 0.159 | 0.001 | 0.002 |  |  |  |  |

*, P<0.05; **, P<0.01; 1-4, 2012-2015.
